# Supplementary material for: Many, but not all, lineage-specific genes can be explained by homology detection failure
Source: PLoS Biol. 2020 Nov 2;18(11):e3000862. doi: 10.1371/journal.pbio.3000862 (PMC7660931; doi:10.1371/journal.pbio.3000862)
Supplement: S1 Supporting Information — Justification for the functional form of the model; effect of site-specific selection pressure; analysis of data in Vakirlis and colleagues (2020). (DOCX) [file pbio.3000862.s013.docx]

**Supplemental Information**

**Model of similarity score as a function of evolutionary distance**

Our model assumes that a similarity score *S* between two homologs is proportional to the number of sites that are identical in these two homologs. After the homologs diverge from their common ancestor, we assume that they undergo a substitution-only mutation process, in which each site in the proteins mutates into a non-identical site at the same protein-specific rate *R* per unit of evolutionary time. Neglecting the possibility of reversion, the probability that a site will *not* undergo a mutation within a time *t* following homolog divergence is e*^-Rt^*, as given by the Poisson distribution. Given a constant number *N_0_* of total sites in the protein, the number of sites that remain identical at that time *t* is binomially distributed with mean *N_0_e^-Rt^*. If each identical site contributes the same amount *c* to the total similarity score, then the mean similarity score at time t is *S(t)* = c*N_0_*e*^-Rt^.* The variance of the similarity score at time t is σ^2^ = c*N_0_*(1-e^-^*^Rt^)*(e^-^*^Rt^*), from the variance of a binomial distribution. In the text, we refer to c*N_0_* as *L*, as these two parameters only appear as a product.

The simplifying approximations in this model abstract away the detailed effects of
the substitution score matrix, insertion and deletion scores, and local versus global sequence alignment. Nonetheless, they appear to suffice to approximate *S(t)* in the regime of observable (i.e. statistically significant) local alignment scores *S(t)* >> 0, although we somewhat underestimate the empirically observed variance, and have some skew toward overestimating *S(t)* (Supplemental Figure 2).

**Modeling site-specific rates via a single protein-wide rate**

Our model of the expected bitscore, *S(t)* = *L*e^-^*^Rt^*, assumes that all sites in the protein evolve at the same rate *R*. As different sites within proteins are well known to evolve at different rates, this is a simplification. However, this model can implicitly capture the effect of these site-specific rates.

For a protein with *L* sites, if each individual site *i* has its own site-specific rate *R(i),* the expected score from each site is *S_i_(t) =* e*^-R(i)t^*, and so the total score for the protein is *S(t) = Σ_I_* e*^-R(i)t^*. We aim to approximate this score with our single-rate model *S(t)* = *L*e^-^*^Rt^*. Our model will capture the effects of site-specific rates to the extent that this approximation can be done well, meaning that a value of global rate *R* that provides a good approximation to full site-specific score can be found.

Qualitatively, the best such value of *R* will be intermediate between the highest and the lowest values of *R(i)*, and weighted toward the lower *R(i)* values (which, since their expected scores decline more slowly, will contribute more to the total score). The quality of this approximation will increase as the *R(i)* values become more similar in value (with an exact approximation of *R*=*R(i)*  in the case where all are identical). Even in the case where the *R(i)* are very different in value, however, the larger *R(i)* values will decline more quickly than their smaller counterparts, contributing less to score and facilitating a reasonable approximation. These features are illustrated in SI Figure 1 below, which depicts the expected scores from individual sites, the total protein score, and the best-fit score from our single-rate model for the simple case of a three-site protein in which the sites span two orders of magnitude in rate.

We fit the scores for a protein to the single-rate model *S(t)* = *L*e^-^*^Rt^*, aiming to find such a value of *R* that minimizes the error between *Σ_I_* e*^-R(i)t^*. and *L*e^-^*^Rt^* and results in a good approximation. Empirically, the quality of the resulting fits indicates that this strategy works well (Supplemental Figure 1).

Simulations also support that such an approximation is generally good. Randomly generating values of *L* and *R(i)* from a uniform distribution bounded by the most extreme values of *L* and *R* observed in our dataset, computing the score *Σ_I_* e*^-R(i)t^,* and fitting this score to our approximating function *S(t)* = *L*e^-^*^Rt^* shows that the error of this approximation, defined as the average value over the range t=0 to t=1, similar to the distances used in our analysis (Figure 2), of the absolute value of the difference between the two functions normalized by the maximum height of the true score (a), is small, clustered around 0.04. Repeating this procedure but for distributions of a and b that are exponentially distributed with means of 2 and 1000 respectively, more closely matching known distributions of protein length and evolutionary rates [37, 38], decreases the error, likely because the average differences between b_i_s decreases.

Thus, our model seems capable of capturing the effects of site-specific rates due to the relatively small error that results from approximating a sum of exponentials with a single exponential.

**Analysis of data from Vakirlis et al. (2020)**

Importantly, we note that Vakirlis et al. did check whether genes in microsyntenic regions were faster-evolving than those outside of these regions. They found that this effect was present and statistically significant (Mann-Whitney p-value=8*10^-5^), but hypothesized that the effect size was likely too small (we compute from their data that the median dN of genes in microsyntenic regions was 9% slower than those without) to affect their results.

This analysis was based on dN values computed from a set of previously-produced alignments that include only 5261 *S. cerevisiae* genes, compared to the 6002 used here. This difference in number is due to two factors: a different underlying *S. cerevisiae* protein annotation, and the inclusion only of genes for which orthologs were identified in all five of the *sensu stricto* yeast species used here. We speculated that this latter factor in particular may impose a bias against faster-evolving genes, for which orthologs are less likely to be detected in all five species. We also speculated that considering dN alone may not comprehensively enough capture the effect of evolutionary rate on homolog detectability, as insertions and deletions are also known to have a large effect.

As a result, we produced our own alignments of all *S. cerevisiae* genes with an ortholog present in at least *S. bayanus* and used them to perform a similar analysis (Methods). These include 400 *S. cerevisiae* genes that are present in our alignments but absent from the previous set of alignments. We used our alignments to compute substitutions/site between *S. cerevisiae* and *S. bayanus* (Methods). For genes in common between the two datasets, the *r*^2^ between these substitutions/site values and the dN values provided by Vakirlis et al. was 0.75. We found that dN values of genes missing from the alignments used in Vakirlis et al were indeed higher than the genes that were included (Mann-Whitney p-value=3*10^-5^), with a clear tail of very fast-evolving genes unique to the missing genes:

However, this effect is driven by a small number of genes. Consistent with this, we find that the overall differences in substitutions/site between genes in syntenic regions and those outside computed from our alignments are similar to those found by Vakirlis et al, excepting the small tail of very fast-evolving genes enriched in the non-microsyntenic distribution not present in the Vakirlis analysis, with a difference in medians of about 9% (Mann-Whitney p-value=9*10^-5^):

We find that the number of gaps in our alignments (normalized for number of positions and number of sequences), representing the number of amino acids that have been inserted and deleted, is on average twice as high for genes in microsyntenic regions than in those outside (Mann-Whitney p-value=3*10^-24^):

Consistent with these two observations, genes in microsyntenic regions have higher values of our R parameter, which incorporates both substitution rate and insertion/deletion rate (Mann-Whitney p-value = 4*10^-12^):

Based on the above analysis, this difference seems to be driven largely by a differential rate of insertions and deletions, perhaps with some smaller contribution from differential substitution rate. We note that although insertions and deletions are commonly not considered in evolutionary rate calculations, their effect on homolog detectability makes them important to bear in mind.

Having noted differences in substitution rate and insertion/deletion rate between the two sets of genes, we asked whether these differences could be impacting the rate of homology detection failure inferred using only genes in microsyntenic regions. Genes in microsyntenic regions are significantly less likely to be predicted to be undetectable in *S. castellii* by our analysis than genes outside syntenic regions (Chi-square p-value=3x10^-13^*),* suggesting that this is the case. (In the below figure, the gray area corresponds to values of *L* and *R* that result in the predicted bitscore being below the detectability threshold of 37, defined by the curve 37 < *Le^-Rt^*, where t is evolutionary distance of *S.* *castellii* from *S. cerevisiae*):

If such a bias were operating, we would expect that, when we restrict our analysis in fungi to consider only lineage-specific genes in microsyntenic regions, we find a much lower rate of homology detection failure compared to the rate inferred using all genes. We find that this is the case when we perform our detectability prediction analysis only on the subset of lineage-specific genes within microsyntenic regions in *S. castellii* (left) and *K. waltii* (right) (Methods). With the caveat that the total number of genes in microsyntenic regions is small (25 and 77 in the two lineages analyzed), around 40% of these genes have values of P(detected | null model) less than 0.05, and around 60% less than 0.5:

This is lower than our findings of around 55% and 75% for all lineage-specific genes. It is also roughly consistent with the estimate in yeast of around 40% from Vakirlis et al.

Based on this analysis, we conclude that these two methods roughly agree when the set of genes used in the underlying analyses are the same.
