# Supplementary material for: Many, but not all, lineage-specific genes can be explained by homology detection failure
Source: PLoS Biol. 2020 Nov 2;18(11):e3000862. doi: 10.1371/journal.pbio.3000862 (PMC7660931; doi:10.1371/journal.pbio.3000862)
Supplement: Attachment — Submitted filename: reviewer_response.pdf [file pbio.3000862.s015.pdf]

Howard Hughes Medical Institute

26 June 2020

Editors, PLOS Biology

Dear editors,

We thank the referees for comments on our manuscript "*Many but not all lineage-specific genes can be explained by homology detection failure*" (PBIOLOGY-D-20-00507R1), and we thank you for the opportunity to submit a revised manuscript. Below we summarize each reviewer's main points and reproduce each of their specific points, with our responses and revisions. We renumbered each specific point in order for ease of crossreferencing.

Reviewer #1:

Reviewer #1 described the work as "*superbly written and reasoned*", "*an important contribution to the field", and "entirely appropriate [...] for PLOS Biology", with "no major issues with the manuscript as written"*.

> (1.) I have been trying to think through whether there are any limitations to using the BUSCO set of proteins to draw inferences about potential lineage-specific proteins, since these classes may differ in some notable parameters. For example, proteins in the BUSCO set are likely to be much longer than a hypothetical, recently evolved de novo evolved protein, to evolve more slowly (since wide conservation is a criterion for inclusion in BUSCO), to have more complex structures, and to perhaps be less prone to duplication. Do the authors think that any of these differences place any limitations on their analyses? I suspect not, since shorter lengths and faster evolution are likely to exacerbate the issue of non-detectability... which just supports the authors' conclusion that these are major limitations to making a claim about novel evolution.

The reviewer is correct that we take advantage of special features of BUSCO genes like length and decreased evolutionary rate: these features make ortholog identification in a large number of species and reliable alignments possible, both of which are essential to our method. However, these differences between BUSCO genes and lineage-specific genes should not affect downstream inference, as the purpose of the BUSCO genes is only to provide a reliable set of relative evolutionary distances between our species. We have emphasized this point in the relevant Methods section.

> (2.) On a related note, one issue that the manuscript highlights for me is the special difficulty posed by protein length in assessing lineage specificity. Even the shortest proteins shown in the examples of Figure 1 are long compared to most of the proteins discussed in studies of novel/de novo genes (e.g., fly eIF4B is 459 a.a.). The graphs in the figure illustrate that the shorter the length, the lower a protein starts on the similarity score y-axis, and thus the less room the score has to fall before it an ortholog of a given distance becomes undetectable at e = 0.001. If the authors see any use in adding a bit in the discussion about how

Sean R. Eddy
Investigator

Ellmore C. Patterson Professor
Molecular & Cellular Biology, and Applied Mathematics
Harvard University, Biological Laboratories 1008A
16 Divinity Avenue, Cambridge, Massachusetts 02138

+1-617-496-6757 | seaneddy@fas.harvard.edu | **eddylab.org**

protein length affects their results and conclusions (e.g., are shorter proteins more likely to fall into the non-detectable category?), it could be interesting, but I defer to their judgement here.

The reviewer is correct: shorter proteins would be, all other things equal, likelier to fall into the undetectable category. We have added more explicit mention of this to the Discussion.

(3.) Did the authors look into how much changing the BLASTP e-value cut-off would affect their results?

Yes. We use E=0.001 in the text, because it has been previously suggested as the standard for BLAST-based lineage-specific gene identification, but we did look into using other cutoffs. In addition to E=0.001, as in the text, we tested E=0.01 and E=0.1. As expected and has been documented before, the number of lineage-specific genes decreases somewhat, usually around ~20% in going from 0.001 to 0.1. The quality of the model fit to data (the distributions of r^2 and of P(undetected | null model) for non-lineage-specific genes) and the distributions of P(undetected | null model) and the percentage of "significant" lineage-specific genes remains approximately constant.

(4.) Another criterion sometimes used to support the de novo emergence hypothesis is that the protein should lack structural similarity to other proteins, as would be expected for a protein that recently emerged from random sequence. This might be useful to consider here, since structural conservation tends to decay less quickly than primary sequence conservation. If there is a good way of assessing this, do proteins that fall into the "should be detectable, but aren't" category (i.e., probability values close to 1 in the top row of graphs in Figs. 4-5) have any major patterns of structural differences from proteins that fall into the "too divergent to be detected" category (probability values close to 0 in these figures)? (If it is easier to look into this specifically for, say, the 25 yeast genes described on p. 9 whose absence outside of the sensu stricto group is poorly explained by homology failure, that would be fine.)

We agree that structural differences of the two classes of lineage-specific genes, and the topic of structural novelty in general, is an interesting topic. We feel that to treat this issue comprehensively is not within the scope of this paper: a proper analysis would likely dilute the current message.

(5.) While the web server for checking individual genes is already very useful, it would also be helpful for the authors to list in a supplemental table the supposedly lineage-specific individual S. cerevisiae and D. melanogaster genes that were used in the analyses for Figs. 4-5, along with their probabilities of detection at each outgroup species "t." This could make it easier for other groups to contribute to the further characterization the manuscript calls for, the genes whose lineage-specificity cannot be easily explained by homology detection failure could be prioritized.

Agreed. We have now included this as Supplemental Table 3.

Reviewer #2:

Reviewer #2 felt that the paper addresses an "*old question*" while failing to "*carefully [look] at underlying assumptions and possible artefacts*" like other papers that suffer "*the curse of the genomic era, that algorithms and statistics dominate the*

outcome". The reviewer "*recommend[s] rejection at this stage*" because of the "*odd way*" in which the manuscript is written, and a"*failure of providing relevant data*" that "*compromise[d] proper reviewing*", such that "*a full evaluation of the specific claims is not possible*"; furthermore, the reviewer felt that even if their concerns were addressed, the manuscript would be "*a better fit into a journal specialized on molecular evolution or algorithm development*". On these overall points, we note that the other reviewers comment positively on the writing of the manuscript and the elegance of the analysis, and we note that we provided all raw data and scripts necessary to reproduce our analyses in supplementary material, on GitHub, and in a webserver linked in the paper.

> (6.) The idea of a null model for the evolution of proteins with a constant substitution rate over time goes back to Zuckerkandl after doing the first globin sequence comparisons (if I remember right). It sparked the question of the possible existence of a molecular clock, which was extremely controversial for some time. The discussions resolved into the conclusion that there is apparently indeed a set of proteins that follow clock-like patterns, but that there are also others that behave rather differently, with lineage-specific changes in substitution rates. Further, it has become clear that whole lineages of taxa can show accelerated substitution rates. In molecular phylogeny studies, it had therefore become standard to test clock assumptions before using a gene for a phylogeny, since one would otherwise deal with artefacts. Hence, the assumption of the present paper that a constant decay rate, evenly spaced across a protein, would be a suitable null-model is an idealistic concept, which is only partially supported by the available data. There were therefore good reasons why previous papers on this question have taken more complex parameters into account (as cited). Why one would want to fall back behind these standards is not clear.

We do not assume a molecular clock. By directly calculating the substitutions per site between pairs of species, we allow for different lineages to evolve at different rates. This is evident in Figure 1, which depicts evolutionary distances incompatible with a molecular clock assumption (e.g. Y. lipolytica, which is topologically a closer outgroup to *S. cerevisiae* than is *S. pombe*, is further in substitutions/site). We have clarified this in the main text of the manuscript where we discuss the computation of these pairwise evolutionary distances.

We do assume that each individual protein's evolutionary rate (relative to other proteins) remains constant over time. We must make this assumption, as our model is a null hypothesis whose aim is to test for deviations from exactly this assumption. We use several tests to determine whether this assumption is accurate enough for our model to usefully detect deviations from this assumption. We find that this is the case, as is shown in Figures 4 and 5 (middle panels), Supplemental Figure 1, and Supplemental Figure 2, which show that fit to the model and the accuracy of our detectability prediction is very good for almost all genes. We have clarified this point in discussing our model.

> (7.) In the present paper, a lineage specific acceleration has not been considered. Diptera (e.g. including Drosophila) show lineage specific acceleration rates, while beetles (e.g. including Tribolium) show particularly low rates (Savard et al. 2006, BMC Evol Biol. 25;6:7). Hence, rate calculations obtained from

> fly comparisons cannot be directly projected to other insects without correcting for this effect. The yeast dataset would also have to be checked for this potential problem.

As mentioned above, our methods do not assume a molecular clock, and do capture the effects of lineage-specific rate accelerations. For example, the low rate in *Tribolium* mentioned by the reviewer is reflected in our results: Figure 2 shows that *Tribolium* has a lower evolutionary distance from D. melanogaster than three other species that are closer in branching order, consistent with a rate deceleration in the beetle lineage. We use the same method in yeast and insects: we also do not assume a molecular clock in yeast. Throughout, we explicitly use pairwise distances in units of substitutions per site, not time, to avoid any assumption of a molecular clock.

> (8.) Second, it is also important to check whether there are even or uneven substitution rates along the length of a given gene, i.e. whether it includes a domain with high conservation in a background of low conservation (for example, most transcription factors and receptors fall into this class). Since BLAST requires only a small seed sequence for detecting a homologue, a short conserved domain is often sufficient to find it, even though the E-value may be pretty low, because of the divergence around it. This was the key argument of Alba and Castresana (ref 24) and it is rather unclear why the current authors throw it over board. The claim that it is better to make more simple assumptions may be appropriate in physics, but is seldomly correct in biology.

Several reviewers raise a similar point. Importantly, our model does not assume position-independent substitution rates along the protein; the reviewer is right that if it did, it would be a poor model of BLAST detectability. We only assume constant (position-independent) rate across a protein sequence to obtain an exact derivation of our model's key equation, but the model will hold approximately for real protein sequences. Our model's abstract protein-specific rate parameter R subsumes and approximates various effects of complex and realistic selective pressure on time-dependent decay of BLAST scores, including position-specific conservation and insertion/deletion patterns, because this parameter is empirically and directly fitted to the observed decay of pairwise BLAST scores. The main mathematical assumption of our model is only that this decay (even with real selection pressures) is approximately exponential. Empirical results presented in the paper support the validity of the model on real protein sequences, including goodness of fit tests presented in Supplemental Figures 1,2.

We made revisions to clarify this important point. A revised paragraph now reads: "Although an exact derivation of this function makes unrealistic simplifications of a substitution-only process with constant position-independent substitution rate, the same functional form will approximate the effects of site-specific rates (Supplemental Information) and position-specific insertion/deletion. Whatever the detailed position-specific selection pressure on the protein is, if that selection pressure is constant over time, we expect similarity scores to decline roughly exponentially. We can empirically estimate that decay curve by fitting L and R to observed scores at different divergence times. By subsuming the complex effects of selective pressure into a two-parameter empirical model of similarity score decline, we avoid the need for a parameter-heavy model of sequence evolution. Vastly reducing the number of parameters in our model allows us to apply our model to genes with a limited number of identified homologs (genes specific to very young lineages) while minimizing problems of results being sensitive to parameter

estimation in complex models. The assumption that rate *R* is the same across evolutionary time and in all lineages is also clearly a simplification, but this is the null hypothesis that we aim to test: we aim to identify genes where a lack of detected homologs is consistent with a constant expected decay of similarity scores with time, without any need to invoke lineage-specific appearance or rate shifts."

> (9.) The few examples they show in the paper cannot convince, since these are picked examples - while the primary data for all analyses are not provided. I expect that one could also pick different examples from them that would show a different pattern.

We showed results from such a goodness of fit test for three example genes in each taxon (Figure 1) for the purpose of illustration. But in addition, we performed this same goodness of fit test for *all* genes in both taxa, and reported the results in Supplemental Figures 1 and 2, which show that the model fits well. The raw data for these figures are available on GitHub and the web server linked in the paper.

> (10.) Further, the use of BUSCO genes for calibration (without providing the relevant primary data) is rather one-sided, since this is a very select group of genes (and anyway usually used for other purposes, i.e. it is unclear why rate validation could be based on it).

The purpose of BUSCO genes in our method is to estimate *relative* evolutionary distances between pairs of species. That BUSCO genes are a select group of genes is our motivation for using them: to estimate accurate relative distances, we must use genes that are single-copy in all species, for which orthology can be readily determined, and which are slow-enough evolving that accurate alignments are possible. These unique features of BUSCO genes do not bias our downstream analysis, as their only purpose is to obtain relative evolutionary distances between species. We describe these motivations in the Methods, and have added clarification there.

> (11.) Another problem that has been extensively discussed in the past is whether the routines for gene annotation generate biases on their own, which in turn bias conclusions drawn from them. There are actually many papers that show this now, starting from the insight that a filter on minimum ORF length does not make much sense, to the realization that quite a few coding regions include more than one functional ORF. Also, annotators consider an annotation as less reliable when no homologs are found in other species and tend therefore to remove them. Hence, when one relies on the annotated list of proteins only, especially on secondarily "curated" lists as it is done here, one loses many of the novel genes. Accordingly, suggesting general fraction-numbers for the relative detectability (or non-detectability) of novel genes does not seem appropriate when one has a biased dataset from start.

We added an analysis that includes genes marked in the Saccharomyces Genome Database as of dubious coding status, which have been removed from the existing RefSeq annotation (Supplemental Figure 5). These include many short and non-conserved genes of the type mentioned here. The result is that an even larger proportion of these genes are predicted to be undetectable, compared to those in the existing RefSeq annotation. We otherwise focus on

existing annotations produced through standard means because most papers studying novel genes take this approach. We agree that it is important to note this explicitly, and now do so in the Discussion.

> (12.) Yet another old discussion is the question of proper alignments to calculate substitution rates. In the early times when people have started to do this, there were clear recommendations that every alignment had to be manually inspected, that indels had to be treated in a consistent way (usually removed throughout the alignment together with some flanking regions) and that length changes needed to be considered etc. In fact, producing an appropriate alignment was a major achievement on its own. The present paper just uses a single algorithm (MUSCLE) and runs it under default parameters only, apparently without further manual inspection. However, it is well known that different parameters need to be used for different proteins, especially the gap penalty parameter can make a huge difference for the substitution rate calculation derived from such an automatic alignment.

We only perform multiple alignments of BUSCO genes for the purpose of producing evolutionary distances between species. Because of this and other concerns about these distances being dependent on a particular alignment, we considered alignments from three different sets of genes in each taxon. We showed that our results are robust to this choice (Supplemental Table 1, Supplemental Table 4). We also show that the fit of the data to our model, parameterized by distances produced by these alignments, is good (Supplemental Figure 1 and 2, Figure 4). Neither of these results would be expected if the underlying alignments provided inaccurate evolutionary distances.

> (13.) If one removes the quantitative message from the paper, there is still a credible qualitative message, namely that a set of genes diverges beyond recognition because of fast evolution, while other genes evolve more slowly, yet show no matches in distant species. This is an important conclusion, but merely confirms what has been shown before (ref 5). These previous authors had basically asked the same question, came to the same qualitative conclusions and even the discussion is similar.

We disagree. The referenced paper, and other previous work that we cite, assumes that genes that lack detectable homology in outgroup species are evolutionarily novel. This is the key assumption that we test here: whether (and how often) genes can lack detectable homology despite the absence of any changes in evolutionary rate caused by a new function.

> (14.) Finally - and this has also been an active discussion in the past years - the authors fail to distinguish between novel genes and de novo evolved genes.

We introduce and define the two classes of genes separately (Introduction), treat the two categories separately in the analysis (Characterization of yeast lineage-specific genes that are poorly explained by homology detection failure), and discuss both classes separately in the Discussion.

> (15.) The papers and reviews on de novo genes from the recent years make it very clear that a proof for de novo evolution can only come from comparisons between very closely related species, where synteny with

ancestral non-coding sequences can be shown. The question of detectability by BLAST is not an issue in these cases, since the genomes to be compared should me >90% similar anyway. Hence, the present paper adds at best speculations to this discussion.

We agree that synteny analyses are the gold standard for assessing claims of de novo evolution. However, such analyses are often not possible, as they require the species under consideration to be closely enough related that synteny is conserved, which is true for only a limited number of taxa. As a result, it remains common for analyses of more distantly related taxa to rely heavily on BLAST, and we cite several recent examples. We have made revisions to the Discussion to make this point clearer.

(16.) The discussion on novelty is also problematic, since it is based on the assumption that two proteins that have diverged beyond recognizability would still have the same function since they are homologs (i.e. have a common evolutionary descent). But, for example, the fore limbs of all vertebrates are homologous and have nonetheless developed different lineage-specific functions. Homology alone does not imply function and this should apply also to proteins. Hence, the identification of remote homologues by itself does not yield insight into the evolutionary novelty question.

By Ockham's razor, we prefer the simplest plausible explanation for observations. The purpose of this paper is to evaluate when a failure to detect homologs outside a lineage is consistent simply with a null hypothesis of constant evolutionary change under unchanged selective pressure, with no lineage-specific *de novo* origination or large rate shifts. A change in molecular function is an example of "novelty" that could cause an evolutionary rate shift, which could cause our null hypothesis to be rejected, flagging a gene as potentially more interesting than other genes that do evolve in a manner consistent with the null hypothesis. We agree that, in the case of additional, experimental evidence suggesting a novel function (as is clearly the case for forelimbs), the hypothesis of novelty may come to be preferred, but such evidence is almost always not available for lineage-specific genes, making our analysis relevant. We revised the Discussion to clarify this further.

(17.) Actually, it would be much more appropriate if this paper would be framed within the context of the evolutionary origin question of proteins that goes back to the famous 1977 paper of Francois Jacob on "Evolution and Tinkering". A main conclusion in the present paper seems to be that the majority of genes has remote homologs, which implies that they would have arisen already at the times of the origin of life, i.e. the authors would probably support the Jacob scenario. It is up to them how they want to discuss this in the light of proven cases for de novo evolution, but this is actually the real question that they are addressing in their study.

Our results do not address the question of the genesis of genes at the origin of life. The timescales that we consider here are ~1By at most, compared to the ~4.5By to the origin of life. We think the reviewer's point is that genes had to have a de novo origination *somewhere* later in evolution, else ancestors of all proteins would all have to have existed at the origin of life (which seems implausible), and we agree. The question our paper helps address is *when*. Our results imply that standard BLAST-based methods for estimating the age of *de novo* gene origination are often biased toward younger ages because of frequent homology detection failure.

(18.) Overall, with its simplified assumptions, this paper has more the character of an academic exercise than leading us a large step forward. It uses a idealistic concept of protein evolution, develops a corresponding algorithm for testing it and comes up with some interesting observations in detail. However, the overall conclusions are not really new and the conclusions on percentages of detectability are based on ignoring the necessity for parameterization, as well as a limited dataset that has excluded the more interesting genes for this question from start.

As explained above, we disagree. We made revisions to clarify.

(19.) - the separation of figures from the text with a further separation of figure legends from the figures makes the paper very difficult to review unless one prints it out; this is a bit outdated
- no line numbers are included, which makes it difficult to list specific comments; hence, although I would have many, I do not add them here, since the paper would anyway require a major revision

We followed the journal guidelines for submission format. We apologize for the inconvenience to the reviewer.

Reviewer #3:

Reviewer #3 said the paper is "*thoughtful and very well written, presenting a novel and useful new methodology*" and was "*happy to suggest that it be accepted by PLOS Biology with some minor revisions*".

(20.) It strikes me that readers may be surprised by how well the proposed neutral model, which simply accounts for length and divergence time/evolutionary rate, fits the data, without accounting for any other forces, e.g. selection to retain function, etc. Some discussion of neutral divergence and the evolutionary forces acting at the protein level could be included in the introduction or discussion.

Our model is not a neutral model. It is an abstract model of the time-dependent decay of pairwise similarity scores. Scores for more conserved proteins decay more slowly than less conserved protein (e.g. Figure 1). The model's rate parameter *R* is fitted specifically to each protein. By empirically fitting the observed decay rate of pairwise similarity scores to each protein, the *R* parameter abstractly includes the effect of various selective forces on protein sequence evolution, including position-specific substitution and indel rates. We have clarified this point in the section discussing our model and in the Methods section describing the purpose of the evolutionary distances. See also the response to point (8) above.

(21.) On a similar note, possibly outside the scope of this work, I wonder if different categories of genes, or genes general to many lineages as opposed to being lineage specific, fit the null model equally well?

We agree that this is an interesting question. We were unable to find any particular correlates of model fit beyond one that we now note in the caption of Supplemental Figure 1: a peak of genes with r^2 close to 0 is comprised of

genes only identifiable in a very closely related group of species, such that their sequences are almost entirely identical throughout, except for a single large deletion event (either misannotation or a true deletion). This results in almost none of the variance in score (of which there is none, save this event) being explained by divergence time. We consider this to be an artifact of the method rather than a biologically meaningful result, as it only appears in the limited cases where the sequences in question are almost totally identical.

> (22.) I would like to see more discussion of the effect of differences in evolutionary rate over time, and over different parts of the protein- especially since the model fit for b is worse to the real gene-specific evolutionary rate.

We added more discussion of why we assume no changes in evolutionary rate over time to the main text and supplement where we discuss our model, as well as a discussion of the difference between the mathematical derivation of our model (where we assume position-independent substitution rates) versus using the model's functional form as an empirical approximation, fitted to the observed decay of pairwise similarity scores of real protein sequences subject to complex selective pressures. We have also added a discussion of the imperfect correlation between the *R* parameter and the evolutionary rate in substitutions per site to the text. See also the response to point (8) above.

> (23.) Results page 16- I find the gene ontology enrichment results difficult to interpret, given that there is no comparison to GO enrichment searches for genes that are not poorly explained by homology detection failure.

Good point. We have added the results of a corresponding GO analysis for genes poorly explained by detection failure and thank the reviewer for pointing this out.

> (24.) discussion- vakirlis et al find evidence that considerably fewer genes are missed due to homology detection failure than the authors. Can the authors expand on this discrepancy further? I found it quite surprising, especially since these Vakirlis et al. also use yeast and drosophila.

We looked into this detail and we think we understand the quantitative discrepancy now. We have moved the discussion of the Vakirilis paper to the Results section and have added discussion of a hypothesis and supporting data regarding the cause of the discrepancy.

> (25.) Suggestions for clarity: - Notation: the authors use a and b for their model parameters. These seem somewhat arbitrary choices, I suggest replacing them throughout: for example, replace 'a' with 'l', for length and 'b' with something like 'r', for rate, in order to help readers follow and distinguish between them.

We have made this change (to *L* and *R)* and we thank the reviewer for the suggestion.

(26.) - Notation formatting inconsistency: e is not constantly italicised throughout the manuscript.

We meant to italicize only variables (S, t, L and R), so have un-italicized e throughout for consistency.

(27.) - Fig 1: the legend could include an overview of what is meant by similarity score, for completeness. The dashed line also appears to be a different width in a than in b, the line in 1A should be made finer, so it is more clearly not at 0.

We have changed the figure accordingly. We were unable to find a way to re-define similarity score that is brief enough as not to overwhelm the content of the caption, but have added a note that directs the reader to the text of the manuscript for a definition.

(28.) - Figure 4 and 5- the authors could consider rearrangement? Following the text as currently written makes the figure layout quite confusing. Rearrange to appear in same order as referenced in text, and/or include slightly longer, more descriptive figure legends to explain the middle row as a kind of positive control, and bottom to more fully explain that the highlighting is for the lineage specificity of genes being included. The focal outgroup species name could also be highlighted in the phylogeny for additional clarity.

We have lengthened and clarified the figure legends and added shading to the focal species name in the phylogeny.

(29.) -Introduction page 9 - inclusion of large divergence in introductory paragraph- this is potentially confusing and could be clarified, because the meaningful distinction between this kind of divergence and genes for which we fail to find homologs is not obvious. If we can't find a homolog, is that sufficient divergence for the evolution of new function?

We aren't sure precisely to which place in the text the reviewer is pointing, as the introduction does not extend to page 9. If this is in reference to the distinction between what we term the novelty hypothesis (that genes with no detectable homologs must be novel, even if they have technical evolutionary homologs elsewhere) and our null hypothesis, we revised to clarify this point (second/third paragraph of introduction).

(30.) - Results paragraph beginning 'As both the sensu stricto yeasts and the drosophilid flies are relatively young lineages': the authors could more clearly lay out their methodology, of including genes specific to lineages with older divergence times in their analysis. The current language of 'testing two additional lineages', seems a bit too brief and may be confusing.

We clarified the methodology in the indicated paragraph.

(31.) - Method page 12 : 'Proteins in the other yeast and insect species' - this makes it sound like insects were included in the yeast analysis, which is not the case?

We changed the wording to avoid this confusion.

(32.) 'Proteins in the other yeast and insect species satisfying this reciprocal best hit criterion were considered orthologs of the S. cerevisiae and protein' - This is quite unclear, 'protein' meaning the protein in the other yeast proteomes? I think generally this methods paragraph could be made clearer- possibly by breaking this section up into two paragraphs, with one describing data collection and ortholog searches, and one describing the reciprocal best BLAST hits methodology.

We corrected the typo (extraneous "and") caught by the reviewer and clarified this section.

Reviewer #4: [identifies himself as Arne Elofsson]
Reviewer #4 comments on the "*very nice mathematical model*" that "*provides some valuable insights*"; he finds the results "*not surprising*", but thinks the paper "*should be published*" if only to "*stop still appearing papers assuming that homology detection is very good*".

(33.) One underlying assumption in this study is that an entire gene has a uniform evolutionary rate. This is certainly not a correct assumption, even it for normal genes might be an acceptable assumption. For globular proteins the variation in evolutionary rates the general trend for variation in evolutionary rate between sites is dominated by residues being buried or exposed. However, here the authors focus on genes that for some reason appears to evolve very fast and certainly they are quite different. Quite many of these proteins are intrinsically disordered and this (in addition to a faster evolutionary rate) means that the ration between insertions/deletions vs mutations is different and that the amino acid preferences are different. I would assume that taking site specific evolutionary rate into account would not shift the results significantly (likely a small number of the remaining potentially de novo created genes remains).

We don't assume this, and made revisions to clarify. See the response to point (8) above. Additionally, r^2 values from that analysis for lineage-specific genes specifically are also good, with a mean of 0.87 compared to 0.85 for all genes in insects, and a mean of 0.86 compared to 0.92 for all genes in fungi). This is evidence that lineage-specific genes in particular do not have features that make our model less able to capture their decline in similarity.

Reviewer #5:
Reviewer #5 says the model is "*simple and elegant*", and the paper "*very clearly written*"; a "*solid contribution*" that "*could prove very useful*".

(34.) The issue of homology detection failure is an important one and has been posed before in the literature. While the authors cite the relevant papers, the manuscript does not sufficiently address how this simulation approach differs from previous ones, why it is better, and to what extent it finds the same or different results. In particular, the simulation approach by Moyers and Zhang was based on a similar premise and was applied to the same two focus species. it would be important to fully consider the similarities and differences between the approaches and compare the results. Relatedly, the introduction is

surprisingly short and does not paint a full picture of the state of the art in the field. Key studies are only mentioned in the results and in the discussion. The manuscript would be greatly improved in the introduction were more complete. What previous studies have addressed the same question? What did they find and how? What evidence is there, apart from absence of similarity, for de novo gene emergence?

We expanded our discussion of previous work in the field of assessing homology detection failure, and moved it from the results/discussion sections to its own dedicated part in the introduction.

(35.) Furthermore, the dichotomy presented by the authors in the second and third paragraph can be misleading since rapidly evolving duplicates do have homologues, while de novo genes do not. It should be explained more clearly, and put in the context of previous literature which grouped genes with homologues together and contrasted them to de novo genes (eg, Vakirlis et al elife 2020).

In the revised Introduction, we clarified the difference between our novelty-free scenario and either *de novo* origination or neofunctionalization.

(36.) Another issue with context concerns what is known about "new" genes. For instance, multiple reports have suggested that they have a higher evolutionary rate than other genes. This is a key piece of context that could drastically impact the validity of the model. Indeed, with the authors approach, such genes will very often, possibly almost always, be explained by the null hypothesis when they are in fact truly "novel". This issue is hard to overcome an should at minimum be acknowledged and discussed in detail as it appears a to be an important limitation.

It is indeed possible for a gene to be consistent with our null hypothesis yet nonetheless be a "novel" lineage-specific gene: i.e. no homologs occur outside the lineage and even if there were, they would be undetectable. Failure to reject the null hypothesis does not mean it has been proven. Rather, our argument is that it would be prudent to conclude that lineage-specificity alone has not established that such genes are novel, because the null hypothesis offers a simpler competing explanation for their apparent lineage specificity. Our method does identify a substantial class of lineage-specific genes where our null hypothesis is rejected (if homologs outside the lineage exist, they would have been found), which suggests that it is not the case that "such genes will… possibly almost always be explained by the null hypothesis", though it is true that some may. The ability to distinguish lineage-specific genes into these two classes (consistent or not with homology detection failure) is an important feature of our approach. We made revisions to clarify this in the Discussion.

(37.) The authors need to provide solid evidence that their model can truly distinguish between an evolutionary rate that is fast, but constant (null model), and an accelerated rate (novelty model). This would also be helpful to address the above.

This is related to the point above. Restating: our model predicts the evolutionary distance at which homology searches are expected to fail, for a given protein, when the evolutionary rate of the protein is constant (relative to

overall lineage-specific divergence rate) (Figures 1, 3, 6). When our model predicts that a fast-evolving lineage-specific gene would not have any detectable homologs outside the lineage even if they existed, this means that the hypotheses of "novelty" vs. "homology detection failure" can't be distinguished from lineage-specificity alone (Figure 3). In contrast, when our model predicts that a fast-evolving lineage-specific gene *would* have detectable homologs outside the lineage even if they existed (Figure 6), then the lineage-specificity of the gene *does* provide evidence that something "novel" happened, such as a protein-specific rate acceleration or *de novo* origin. There are several fast-evolving genes that fall into this latter category, as can be seen from the supplemental information available here and on our Github. Just one example is S. cerevisiae *REG2*: this gene rejects our null model more strongly than almost any other gene (P(detected | null model) is approximately 1), but is also quite fast-evolving, with an *R* of about 5.5 (the mean over all genes is 2.1, with a standard deviation of 1.6).

> (38.) The model that the authors propose makes an array of assumptions. For example, we understand that since the predicted bitscores correlate well with the real ones, it's safe to use them. However in the insect dataset there is plenty of variability and a peak seemingly at 0 (supp fig 1). This is important because it shows that, at least in the insects dataset, there are many cases where the model doesn't fit. This of course is expected; after all no model is perfect. But the authors should make sure that there isn't anything particular about these genes that makes them deviate from the model (and whether this could be relevant to lineage-specific genes). This caveat should also be appropriately discussed.

The peak of genes with r^2 close to 0 in the insect dataset is comprised of slowly-evolving proteins only identifiable in the very closely related drosophilid flies. Because of the short timescale and slow evolutionary rate involved, these proteins are almost entirely identical in sequence, except for a single large deletion event that occurs in a species at intermediate evolutionary distance (which may be either a misannotation or a true deletion; many coincide with whole exons, which makes misannotation seem likely). This results in almost none of the variance in score (of which there is none, save this event) being explained by evolutionary distance.

We consider this an artifact of the method, as it only appears in the limited cases where a) the sequences in all identified orthologs are almost totally identical, and b) there are enough such sequences that a large score-affecting event like a deletion/misannotation has a reasonable chance of occurring. We suspect that this is why the peak is specific to insects and does not appear in fungi, where there are only 5 species of similarly high relatedness vs. the 12 drosophilid flies.

We added an explanation of this to the figure caption.

> (39.) The source data for each gene should be made available.

All source data were made available on the GitHub and the web server linked in the manuscript.

> (40.) Another assumption made is that evolutionary rates are stable across sites; but this is widely known not to be the case. This is another important limitation and should be acknowledged.

We don't assume this, and revised to clarify; see response to point #8 above.

> (41.) Synteny guided similarity searches are presented in two parts in the manuscript. First, for those genes for which homology detection failure is a very likely explanation. In this part, we read that out of the 126 S. cerevisiae genes considered, only 24, so 1/5 have an orthologous locus in at least one outgroup, based on YGOB. First, it should be clear whether that means simply that the syntenic region is identifiable or that there is also a gene present. Given the number of comparisons and the level of conserved synteny in yeasts the former would be surprising. If the latter, the number of cases where the orthologous region can be identified but no gene exists should be provided and commented upon. This is an important control for the authors' main claim: given that these genes are the best candidates for having simply diverged beyond detectability at the level of the clade in question, we would expect that for many of them a candidate homologue would be present in the predicted genomic region. If this isn't the case the authors should discuss why.

First, this number of 24 genes should actually be 19/126 genes (a typo that resulted from conflation with the synteny bitscore cutoff of 24; we thank the reader for causing us to catch this, and have corrected it in the text).

The former is the case: these 19 genes are indeed the only ones of the 126 which have any orthologous locus listed in YGOB. Many of the other genes are not included in YGOB at all (although they are not marked as of "dubious" coding status, many are termed only "putative" genes and so have not been included). We have clarified this in the text.

Of these 19 genes with an identifiable orthologous region, 17 have an annotated gene in an outgroup at the locus, while only 2 do not. We have added this figure to the text as requested.

The fact that nearly all genes do have an annotated gene in an outgroup is consistent with the hypothesis of divergence beyond detection, as the reviewer suggests. However, in the other two cases, the absence of such a gene at the locus may be merely due to annotation failure, as we note in the text: we do not attempt to find unannotated ORFs with significant similarity at the locus.

That the outgroup gene at the orthologous locus shows significant similarity for only 11 of these 19 genes is again consistent with divergence beyond detection: as mentioned in the text, our predicted similarity score for all of these 19 genes is below the synteny detection threshold of 24.

> (42.) Second, for those genes for which homology detection failure seems very unlikely. In this part, the out of lineage orthologues are not provided in a supplementary table, as is the case previously. The authors should make these data available.

We added this information to supplemental table 6.

(43.) By design the analyses only focus on the most conserved subset of lineage-specific genes: those with sufficient homology to be able to fit model parameters. Moreover, yeast dubious ORFs are not included although they have been proposed to be enriched for true evolutionary novelty. This should be discussed as it is context for interpreting the main findings of the manuscript, which in several places reads as if it could provide an estimate of what proportion of lineage specific genes are truly novel. In fact, the manuscript estimates proportions based on a restricted search space (that which is conserved enough for the model to be applied). More careful attention to the writing of these conclusions, including in the title, is requested. Note that this limitation to the search space could potentially also explain why the estimates are higher than in previous literature such as Vakirlis et al. 2020, a question raised in the discussion.

We added an analysis of dubious yeast ORFs (Supplemental Figure 5) and explained that the motivation for this is that such ORFs have been previously hypothesized to include novel genes. This analysis shows that an even larger proportion of these genes (nearly all) are predicted to be undetectable than is the case for genes in the existing RefSeq annotation.

We also agree that it is important to highlight the set of genes that is under consideration and the potential limitations therein, and have now explicitly addressed this in the Discussion.

The Vakirlis 2020 paper also uses the NCBI annotation, which does not include dubious ORFs, and so this is unlikely to contribute to the discrepancy. We have done a deeper study of the differences between our results and Vakirlis', which we included (Supplemental Figure 6). The subset of microsyntenic orthologous genes that Vakirlis et al studies, which they assume are representative of all genes, are biased toward longer and more conserved proteins that are more easily detected by homology searches (Supplemental Figure 6b). When we use our method only on a subset of microsyntenic orthologs, we essentially reproduce the same estimate as Vakirlis et al. for homology detection failure (Supplemental Figure 6c).

(44.) Additional questions and minor concerns. - Supp. Tables 2 and 5 are the same file. The legend of Supp. Table 3 does not correspond to what the table actually is. The .eps format for supplementary figures is in general problematic.

We thank the reviewer for pointing out the mistake with the supplemental tables and have corrected the error.

We followed journal submission guidelines for figure file format. We apologize to the reader for the inconvenience.

(45.) Why include a bitscore data point of the alignment of the protein against itself? This is not justified in the manuscript. How does removing this affect the results?

The score of the protein against itself is in our model a score just like any other, representing the similarity score at zero divergence (t=0). In order to produce as accurate a fit as possible, we do not omit any available scores S(t), including this one, when fitting our model.

> (46.) Why is the fungi dataset only half as big as the insect one? Since this is a substantial difference it should be appropriately justified.

The difference in size is due to the difference in the number of proteins in the *D. melanogaster* and *S. cerevisiae* NCBI annotations. We did not alter these annotations before use.

> (47.) Why were orthologues predicted using a method that the authors themselves admit is less than perfect, instead of using pre-computed ones from one of the many available resources of orthologues identified using more sophisticated approaches than RBH (https://questfororthologs.org/orthology_databases) ?

We aim for our method to be easily generalizable to other organisms, including those which have not been previously sequenced or without well characterized genomes. We therefore wanted to demonstrate the efficacy of the method in a way that minimizes reliance on external information and datasets.

> (48.) Some data did not appear to have been made available. For example, the lists of the lineage-specific genes at the various clade levels, the probabilities for individual genes etc. All such data should be made available.

We have added a list of all lineage-specific genes in the six lineages and their probabilities as Supplemental Table 7. Additionally, all raw data, scripts used to produce our results, and all results themselves are available on the linked GitHub.

> (49.) In the supp tables, species names should be italicized.

We made this change.

Again, we thank you and the reviewers for your comments, and we hope our revised manuscript is viewed favorably!

Sincerely,

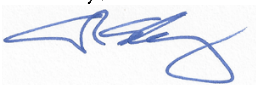

Sean R. Eddy
