# Supplementary material for: Many, but not all, lineage-specific genes can be explained by homology detection failure
Source: PLoS Biol. 2020 Nov 2;18(11):e3000862. doi: 10.1371/journal.pbio.3000862 (PMC7660931; doi:10.1371/journal.pbio.3000862)
Supplement: Attachment — Submitted filename: reviewer_response.pdf [file pbio.3000862.s016.pdf]

hhmi | Howard Hughes Medical Institute

4 August 2020

Editors, PLOS Biology

Dear editors,

We thank the referees for the additional comments on our revised manuscript "*Many but not all lineage-specific genes can be explained by homology detection failure*" (PBIOLOGY-D-20-00507R2). We have made additional revisions to address reviewer #5's remaining concerns as follows:

> … this novel method is a promising addition to the field, and would warrant publication if two major changes are made to the manuscript:
>
> 1 - in the writing of the manuscript (the whole manuscript, from title to discussion), be clear about the limitations of the method, specifically that it can only be applied to subset of genes: those with at least two known homologues within a lineage, and no known homologues outside of the lineage. In the two lineages studied, this is 155 annotated yeast genes (of 375 annotated lineage specific genes), 167 dubious yeast genes (of 784 dubious yeast genes), 1278 drosophila genes (of 1611 lineage specific drosophila genes). These numbers do not justify the generality of claims made by the authors of having applied their method to "all" lineage-specific genes, nor their quantitative and qualitative conclusions reflected in their title and discussion (including that "nearly all" dubious orfs fail to be detected).

We added additional clarifications throughout the introduction and methods to reiterate that we only analyze genes with at least 2 orthologs identified in outgroups, and not all lineage-specific genes. For example, a new sentence in the Introduction reads "The resulting method can be used on any gene with detected homologs in at least two taxa" and revised sentences in the Results read: "We identified all genes specific to each of these four additional lineages, and then calculated P(detected | null model, $t_{outgroup}$) for all genes <u>with the required 2 identified orthologs</u>, exactly as described for the two lineages above." (new text underlined), and "We analyzed the 167 of these dubious genes that met our analysis requirement of having detected orthologs in at least two other species (many are unique to S. cerevisiae)."

We also added this caveat to the Discussion: "Two important caveats should be kept in mind. … Second, these results may or may not generalize to classes of lineage-specific genes that we have not considered here. Because our method requires at least two observed orthologs, we have only applied it here to genes found in at least two outgroup species in the lineages in question."

We disagree that the title of the manuscript makes overly general claims. The title does not contain the word "all." Although we do not analyze all lineage-specific genes, the hundreds to thousands of such genes that we find to be consistent with detection failure makes the word

Sean R. Eddy
Investigator

Ellmore C. Patterson Professor
Molecular & Cellular Biology, and Applied Mathematics
Harvard University, Biological Laboratories 1008A
16 Divinity Avenue, Cambridge, Massachusetts 02138
+1-617-496-6757 | seaneddy@fas.harvard.edu | **eddylab.org**

"many" an accurate descriptor.

> 2 - Given that the method, and its application to individual genes, are the key novel points of the manuscript, the authors need to demonstrate that the method indeed works on lineage specific genes. That is, they need to show that the parameter estimation step of their methods yields accurate results when only two homologues in closely related species are used. This can be done by using conserved genes and estimating the parameters when considering all versus only two closely related homologues, and comparing the results. This needs to be done at large scale, rather than on hand picked examples, in order to quantify the success rate of the method. Without such a quantitative evaluation of the method in the context it is meant to be applied to, we do not know if it really is informative.

To address this concern, we performed the analysis for all genes using only bitscores from orthologs in the two yeast species most closely related to *S. cerevisiae* (*S. paradoxus* and *S. mikatae,* at evolutionary distances of 0.05 and 0.09 respectively). We compared these values to the values of these parameters when computed using available orthologs from all 12 fungi, with the most distant species *S. pombe* at a distance of 0.92. This represents a test of the concordance of these parameters in the worst-case scenario in which a) there are only the minimum required two orthologs available for the parameter fit, and b) those two orthologs come from the two species closest to *S. cerevisiae*, which are most prone to noise due to small numbers of substitutions and capture the least amount of the fluctuation in rate occurring across evolution. (This worst-case scenario is relatively rare in this dataset. In yeast, 1.3% of genes only have two orthologs available to be used in the fit, and for 0.5% of genes are those two orthologs from these two closest species.) The r^2 for this comparison is 0.99 for the L parameter and 0.78 for the R parameter. On average, the value computed from only *S. paradoxus* and *S. mikatae* and the value computed from all species differ by 0.6% for the L parameter, and 8% for the R parameter.

A more informative test of how limited numbers of orthologs affects performance may be direct assessment of whether, from limited numbers of orthologs, the method correctly computes the probability of a homolog being detected. This is the central quantity of interest. To address this question, in each of the six lineages that we consider, for all genes successfully detected in outgroups, we ran our analysis, but artificially limited the orthologs used in all calculations to those from species within the lineage. All bitscores from more distant species were held out of the analysis. We then asked, from these limited data, whether our method correctly predicts that an ortholog is in fact detected in outgroups. Supplemental Figures 4 and 5 show that this is the case. Our method produces probabilities of being detected that are very close to 1 for nearly all genes conserved in outgroups to these lineages.

We have added the result of these additional analyses to the text.

Minor points:

> Several of the modifications and new analyses presented by the authors seem to points to indels/gaps as a potential major limitation of the approach. It may be difficult to address for this manuscript, but it would be nice to point this out clearly in the discussion and present it as an exciting opportunity for future research in the field.

We agree that indel rates affect detectability of homologs (although we do not agree that indels/gaps present a major limitation to our approach). We have added additional discussion of this point to our discussion of the Vakirilis work (now in a dedicated section in the Supplemental Information).

> 34. in summarizing the recent work of Vakirlis et al. the authors mention that the method Vakirlis et al applied does not allow to determine which particular lineage-specific genes may be due to homology detection failure. This statement is false. What is true is that that method cannot determine all of such genes across the genome (it only finds those with adequate synteny). But of course, neither does the authors' approach (it only applies to those with two or more homologues).

We revised the discussion of the Vakrilis work accordingly.

> 36. The issue is not adequately addressed in the discussion. The authors need to clarify that "it is possible for a gene to be consistent with the null hypothesis yet nonetheless be a novel lineage specific gene" if, as suggested by multiple reports, novel genes have high evolutionary rates. The discussion, as well as the title of the manuscript, read as if failure to reject the null hypothesis should be interpreted as the gene is not novel because that is the most conservative assumption. Instead, the percentage of genes that are truly novel but fail to reject the null hypothesis (ie, the false negative rate of the author's method) is unknown. This is a general limitation of the method and should be clearly stated as such.

The Discussion states: "We cannot exclude the possibility that some genes may be truly novel, but are also short enough and evolving fast enough that their orthologs would not be detected if present, such that homology detection failure also appears to be a sufficient explanation for their lineage-specificity. This may be especially salient for de novo genes, which have been widely hypothesized to be short and fast-evolving." In order to draw reader attention to the importance of this point, we revised this text to be more emphatic:

"Two important caveats should be kept in mind. First, this method cannot exclude the possibility that a gene is truly novel, but also short enough and evolving fast enough that its ortholog would not be detected if present. Such genes would be novel but they would also be flagged as genes whose lineage restriction could be explained by homology detection failure. For this reason, it may be difficult for de novo genes, which have been hypothesized to be both short and fast-evolving, to reject the null model."

The central contribution of our manuscript is a method for testing a null hypothesis, that the failure to detect homologs of some protein outside some clade is consistent with when homology searches are expected to fail, given the empirically observed evolutionary rate of the known homologs. When observed data are consistent with both a hypothesis of interest (here: de novo gene origination) and a null hypothesis (here: homology search failure), the data do not support drawing a conclusion in favor of the hypothesis of interest. The phrase "can be explained by" in our title means that homology detection failure cannot easily be ruled out as a null hypothesis for many lineage-specific genes, not that homology detection failure is the *only* possible explanation for lineage-specificity. We make no claim in the paper that says these genes "are due to" or "can only be explained" by homology detection failure.

> 43. This point was not sufficiently addressed in the author's response, so it is is reiterated as the first major comment here.

(We addressed the first major comment above.)

> However, the authors have added an additional analysis about Dubious ORFs which is interesting but presents some important limitation in the statistical analysis of the results and in their interpretation.

We think the reviewer is referring (here and in the remaining points) to the added analysis of the Vakirlis *et al.* approach, not to dubious ORFs.

> Do all genes analyzed have a detectable homologues in S Kud? If not, how can then all the distances be measured relative to S Kud? Then the authors state that "We chose S. kudriavzevii because it is the most distant species from cerevisiae according to our analysis (Figure 2)." But in Figure 2 the most distant Saccharomyces species from cerevisiae is S. bayanus. Is this a typo? Perhaps the methodological explanations can be better described here.

Yes, this was a typo: we used the more distant *S. bayanus* in this analysis, as before. Indeed, all analyzed genes have a detectable homolog in at least *S. bayanus*, as is necessary for this analysis, as the reviewer noted. We have made these corrections and clarifications in the text.

> The conclusions and interpretations are not statistically supported. All comparisons between distributions must be quantified with effect size and p-value in order to make any conclusion.

We added effect sizes and p-values.

> The authors make no mention of the evolutionary rate comparison within and outside of microsyntenic regions presented by Vakirlis et al. It is crucial, if the authors want to support their point, to do a fair comparison and discuss their results in the context of the Vakirlis results. The authors could show their distributions separately for genes also

included in the Vakirlis et al. dataset and those ~400 not included, and do separate statistics for the two groups. This would immediately show if a bias is present in these genes specifically, or if it's a matter of the different measures being used for evolutionary rate.

Vakirilis et al. do in fact report that microsyntenic genes are slower-evolving as measured by dN. We recapitulate this using our new alignments, and there is fairly high concordance between our substitutions/site values and the dN values provided by Vakirilis et al. for genes in common between the two datasets ($r^2 = 0.75$).

However, because the difference in rate between the two groups of genes is higher when measured by insertions/deletions than by substitutions/site, we believe that the bias is driven more strongly by indels than by substitutions.

We added this point to our discussion of the Vakirilis work, now in its own section in Supplemental Information.

46. Our question was referring to the difference in number of species, not number of genes. This question still requires an explanation.

Because many papers on lineage-specific genes have been done in fungi, our main concern was with using species that have been included in these previous studies for the sake of comparison of results. In considering the insect clade, we then aimed to maintain a similar number (7 vs 9) of outgroup species relative to the main, closely-related lineage under analysis (the 12 drosophilids in insects, and the 5 sensu strictus in yeasts). We included 2 additional insect species merely because they were readily available and of high genome quality.

Sincerely,

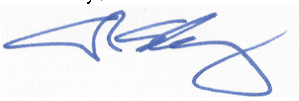

Sean R. Eddy
