# Supplementary material for: Many, but not all, lineage-specific genes can be explained by homology detection failure
Source: PLoS Biol. 2020 Nov 2;18(11):e3000862. doi: 10.1371/journal.pbio.3000862 (PMC7660931; doi:10.1371/journal.pbio.3000862)
Supplement: Attachment — Submitted filename: review_uploaded.docx [file pbio.3000862.s014.docx]

Virtually all genomes contain genes that lack detectable homologues beyond a certain evolutionary distance. There is high interest in understanding the evolutionary mechanisms that underlie the emergence of these lineage-specific genes. In this manuscript, the authors propose to contrast two classes of evolutionary mechanisms: mechanisms that involve “novelty”, such as de novo gene origination or rapid divergence following a duplication event; versus “uneventful” mechanisms, where a steady evolutionary rate eventually leads to the lack of detectability of ancient homologues. The authors present an analytical approach to identify lineage-specific genes that could be explained by a “null model” of homology detection failure due to uneventful evolution at a steady rate. The authors define a simple and elegant mathematical model based on such a scenario and apply it to two lineages, focused on S. cerevisiae and D. melanogaster. They find that potentially a majority of lineage-specific genes could be explained by homology detection failure. The manuscript is very clearly written and has the potential to be a solid contribution to our understanding of the origins of lineage-specific genes and to the wider field of evolutionary genomics. The method itself, freely available on github, could prove very useful for the research community.

Major concerns

The issue of homology detection failure is an important one and has been posed before in the literature. While the authors cite the relevant papers, the manuscript does not sufficiently address how this simulation approach differs from previous ones, why it is better, and to what extent it finds the same or different results. In particular, the simulation approach by Moyers and Zhang was based on a similar premise and was applied to the same two focus species. it would be important to fully consider the similarities and differences between the approaches and compare the results.

Relatedly, the introduction is surprisingly short and does not paint a full picture of the state of the art in the field. Key studies are only mentioned in the results and in the discussion. The manuscript would be greatly improved in the introduction were more complete. What previous studies have addressed the same question? What did they find and how? What evidence is there, apart from absence of similarity, for de novo gene emergence? Furthermore, the dichotomy presented by the authors in the second and third paragraph can be misleading since rapidly evolving duplicates do have homologues, while de novo genes do not. It should be explained more clearly, and put in the context of previous literature which grouped genes with homologues together and contrasted them to de novo genes (eg, Vakirlis et al elife 2020).

Another issue with context concerns what is known about “new” genes. For instance, multiple reports have suggested that they have a higher evolutionary rate than other genes. This is a key piece of context that could drastically impact the validity of the model. Indeed, with the authors approach, such genes will very often, possibly almost always, be explained by the null hypothesis when they are in fact truly “novel”. This issue is hard to overcome an should at minimum be acknowledged and discussed in detail as it appears a to be an important limitation.

The authors need to provide solid evidence that their model can truly distinguish between an evolutionary rate that is fast, but constant (null model), and an accelerated rate (novelty model). This would also be helpful to address the above.

The model that the authors propose makes an array of assumptions. For example, we understand that since the predicted bitscores correlate well with the real ones, it’s safe to use them. However in the insect dataset there is plenty of variability and a peak seemingly at 0 (supp fig 1). This is important because it shows that, at least in the insects dataset, there are many cases where the model doesn’t fit. This of course is expected; after all no model is perfect. But the authors should make sure that there isn’t anything particular about these genes that makes them deviate from the model (and whether this could be relevant to lineage-specific genes). This caveat should also be appropriately discussed. The source data for each gene should be made available. Another assumption made is that evolutionary rates are stable across sites; but this is widely known not to be the case. This is another important limitation and should be acknowledged.

Synteny guided similarity searches are presented in two parts in the manuscript. First, for those genes for which homology detection failure is a very likely explanation. In this part, we read that out of the 126 S. cerevisiae genes considered, only 24, so 1/5 have an orthologous locus in at least one outgroup, based on YGOB. First, it should be clear whether that means simply that the syntenic region is identifiable or that there is also a gene present. Given the number of comparisons and the level of conserved synteny in yeasts the former would be surprising. If the latter, the number of cases where the orthologous region can be identified but no gene exists should be provided and commented upon. This is an important control for the authors’ main claim: given that these genes are the best candidates for having simply diverged beyond detectability at the level of the clade in question, we would expect that for many of them a candidate homologue would be present in the predicted genomic region. If this isn’t the case the authors should discuss why. Second, for those genes for which homology detection failure seems very unlikely. In this part, the out of lineage orthologues are not provided in a supplementary table, as is the case previously. The authors should make these data available.

By design the analyses only focus on the most conserved subset of lineage-specific genes: those with sufficient homology to be able to fit model parameters. Moreover, yeast dubious ORFs are not included although they have been proposed to be enriched for true evolutionary novelty. This should be discussed as it is context for interpreting the main findings of the manuscript, which in several places reads as if it could provide an estimate of what proportion of lineage specific genes are truly novel. In fact, the manuscript estimates proportions based on a restricted search space (that which is conserved enough for the model to be applied). More careful attention to the writing of these conclusions, including in the title, is requested. Note that this limitation to the search space could potentially also explain why the estimates are higher than in previous literature such as Vakirlis et al. 2020, a question raised in the discussion.

Additional questions and minor concerns

- Supp. Tables 2 and 5 are the same file. The legend of Supp. Table 3 does not correspond to what the table actually is. The .eps format for supplementary figures is in general problematic.

- Why include a bitscore data point of the alignment of the protein against itself? This is not justified in the manuscript. How does removing this affect the results?

- Why is the fungi dataset only half as big as the insect one? Since this is a substantial difference it should be appropriately justified.

- Why were orthologues predicted using a method that the authors themselves admit is less than perfect, instead of using pre-computed ones from one of the many available resources of orthologues identified using more sophisticated approaches than RBH (<https://questfororthologs.org/orthology_databases>) ?

- Some data did not appear to have been made available. For example, the lists of the lineage-specific genes at the various clade levels, the probabilities for individual genes etc. All such data should be made available.

- In the supp tables, species names should be italicized.
